# Quantitative Assessment of 3D Dose Rate for Proton Pencil Beam Scanning FLASH Radiotherapy and Its Application for Lung Hypofractionation Treatment Planning

**DOI:** 10.3390/cancers13143549

**Published:** 2021-07-15

**Authors:** Minglei Kang, Shouyi Wei, J. Isabelle Choi, Charles B. Simone, Haibo Lin

**Affiliations:** New York Proton Center, New York, NY 10035, USA; awei@nyproton.com (S.W.); ichoi@nyproton.com (J.I.C.); csimone@nyproton.com (C.B.S.II); hlin@nyproton.com (H.L.)

**Keywords:** proton therapy, pencil beam scanning, dose rate, FLASH radiotherapy, lung hypofractionation

## Abstract

**Simple Summary:**

As pencil beam scanning (PBS) proton therapy delivers doses via spot-scanning, the dose rate quantification is very different from the electron and scattering proton techniques in FLASH radiotherapy. Currently, there is no consensus on the definition of the PBS proton therapy dose rate calculation for normal tissues and targets. This study focuses on the dose rate quantification of organs-at-risk and target based on three proposed dose rate metrics using proton transmission beams. The differences in dose rate metrics have led a large variation for organs-at-risk dose rate assessment and may result in a different correlation expectation between dose rate and biological effects for pre-clinical experiments. An awareness of the differences in proton PBS dose rate calculation is important to design experiments and clinical trials to uncover FLASH-RT’s biological and physiological mechanisms.

**Abstract:**

To quantitatively assess target and organs-at-risk (OAR) dose rate based on three proposed proton PBS dose rate metrics and study FLASH intensity-modulated proton therapy (IMPT) treatment planning using transmission beams. An in-house FLASH planning platform was developed to optimize transmission (shoot-through) plans for nine consecutive lung cancer patients previously planned with proton SBRT. Dose and dose rate calculation codes were developed to quantify three types of dose rate calculation methods (dose-averaged dose rate (DADR), average dose rate (ADR), and dose-threshold dose rate (DTDR)) based on both phantom and patient treatment plans. Two different minimum MU/spot settings were used to optimize two different dose regimes, 34-Gy in one fraction and 45-Gy in three fractions. The OAR sparing and target coverage can be optimized with good uniformity (hotspot < 110% of prescription dose). ADR, accounting for the spot dwelling and scanning time, gives the lowest dose rate; DTDR, not considering this time but a dose-threshold, gives an intermediate dose rate, whereas DADR gives the highest dose rate without considering any time or dose-threshold. All three dose rates attenuate along the beam direction, and the highest dose rate regions often occur on the field edge for ADR and DTDR, whereas DADR has a better dose rate uniformity. The differences in dose rate metrics have led a large variation for OARs dose rate assessment, posing challenges to FLASH clinical implementation. This is the first attempt to study the impact of the dose rate models, and more investigations and evidence for the details of proton PBS FLASH parameters are needed to explore the correlation between FLASH efficacy and the dose rate metrics.

## 1. Introduction

Pre-clinical investigations have shown that ultra-high dose rate (>40 Gy/s) electron beam radiotherapy (FLASH radiation therapy (RT)) leads to fewer radiation-induced toxicities, but is as effective as conventional dose rate radiotherapy regarding tumor control [1,2]. With growing interest in this novel dose delivery approach, recent studies have reported that FLASH-RT achieves enhanced normal tissue protection compared to conventional-RT in the mouse brain, pig skin, and cat experiments [3,4,5]. Fouillade et al.’s mouse and human lung cell in vitro experiments showed that FLASH-RT can minimize the induction of pro-inflammatory genes and persistent DNA damage and facilitates radiation recovery by preserving lung progenitor cells [6]. Bourhis et al. reported the first FLASH-RT skin treatment using a linac with a favorable outcome both on normal skin and tumor [7].

A proof-of-concept FLASH-RT experiment, using a clinical proton system, was performed at the Institut Curie France, where a max dose rate of 40 Gy/s was reached with scattering delivery techniques [8]. Buonanno et al. firstly reported the long-term effects of proton irradiations at FLASH dose rates, in vitro, using a low energy experimental DC accelerator [9]. Beyreuther et al. studied the FLASH effect through irradiating zebrafish embryo using the scattering technique on an IBA proton system [10]. More recently, researchers from the University of Pennsylvania reported their FLASH progress on an IBA proton system, where a dose rate > 100 Gy/s was reached on a small animal radiation therapy platform via scattering systems [11]. The effects of FLASH irradiation using pencil beam scanning (PBS) proton irradiation in a Varian ProBeam system were then reported by Cunningham et al. [12]. 

Proton therapy techniques have been identified as potential platforms for the clinical translation of FLASH-RT [13,14]. For current proton PBS planning and treatment, multiple energy layers are used to generate spread-out Bragg peaks (SOBP) to cover the target volume. However, using SOBP becomes difficult for FLASH-RT to deliver ultra-high dose rate spots across an entire target volume with sufficiently high mean dose rates due to the inefficiency of beam transmission for lower energies beam [13,15]. Additionally, the typical energy/layer switch time is ~200 ms for energy degradation-based cyclotron systems [16] and on a scale of >1000 ms for synchrotron systems [17], which also prolongs the beam-on time. Therefore, the current intensity-modulated proton therapy (IMPT) planning strategies using multiple energies hardly reach the FLASH dose rate threshold in OARs [18]. To date, there are no in vivo data for conformal FLASH irradiations from any proton PBS system using SOBP treatment planning [13], while transmission delivery using beam shoot-through from different angles with a single high-energy is more practical to reach the FLASH dose rate and also minimizes range uncertainties in heterogeneous tissues like the lung.

Unlike electron and proton scattering techniques that deliver uniform fluence to the entire field simultaneously, PBS requires hundreds of pencil beam spots to be delivered sequentially to cover the entire target volume. Due to its intrinsic nature, the PBS dose rate quantification is much more complex. Most clinical proton systems use cyclotrons to accelerate charged particle beams via a high frequency (MHz) alternating voltage, and protons are accelerated to the desired energy and then extracted by an electrostatic field. Protons are concentrated into bunches as quasi-continuous current during the delivery. The instantaneous beam current can be specified as the mean current of each pulse. The magnitude of the instantaneous proton current can be adjusted by the cyclotron control system, which correspondingly changes the instantaneous dose rate in the treatment nozzle. The lateral spot dose profile usually follows a Gaussian distribution in the air or homogeneous phantom. Thus, the center of the spot has a maximum dose rate, and the dose rate decreases radially from the center to the lateral direction of a spot. In our TPS, we generated a single spot plan and placed the isocenter at the water phantom surface, the max dose rate at ~4 cm of the central axis in the water phantom was defined as the spot peak dose rate (SPDR) [19], which was used to compare the dose rate for different minimum MU/spot settings. The spot size is an important factor determining the rapidity of the transverse dose rate falloff for a given spot, while the scanning speed between spots determines the averaged (mean) dose rate. It will be crucial to consider all of these factors to assess if the normal tissue or OARs reach the dose rate threshold for the desired FLASH sparing effect.

To date, there are two distinct proposals on the calculation of dose rate of PBS proton beam: one is weighing the dose rate by the spot dose contribution to an individual point-of-interest dose-averaged dose rate (DADR) [18], and the other method is to average the dose deposition in a region-of-interest over time—averaged dose rate (ADR) [20]. Recent studies also indicate that the FLASH sparing effect is not only determined by dose rate but also related to the dose. Bourhis et al. summarized all of the most relevant parameters for the FLASH effect and concluded that those parameters are “the combination of dose, dose rate within the pulse, and overall time of irradiation (<200 ms), and not only the mean dose-rate as we initially thought.” [21]. All pre-clinical studies to date [1,3,11,22,23] have been performed using a pulse structured beam and dose rate characterized by (mean) instantaneous dose rate; thus, the instantaneous dose rates of the spots can be an important indication to correlate the FLASH sparing effect. It might be more relevant to use a dose-threshold to exclude the PBS low dose tails that deposit doses less than a dose-threshold from the instantaneous dose rate calculation for a region-of-interest (voxel). Therefore, we proposed a dose threshold dose rate (DTDR) to quantify the 3D dose rate distribution for FLASH irradiation. In the hypothesis, a dose rate larger than the currently accepted FLASH-RT threshold (40 Gy/s) is expected to optimize FLASH treatment planning and biological experimental designs [19]; however, it is not clear how the different dose rate calculations may affect the treatment planning considerations and biological and clinical outcomes. On the other hand, the underlying biological mechanism of a FLASH effect remains incompletely determined. Multiple hypotheses have been suggested by linking the high dose rate to the rapid oxygen depletion [24,25], immune response [26,27], reduction of peroxyl radical lifetime [28], preservation of normal tissue stem cells [22,29], etc. The quantitative assessment of the different dose rate methods will be meaningful to give guidance for FLASH-RT planning and treatment. This study aims to investigate the FLASH-RT dose rate determination and its impact on treatment planning toward clinical applications.

## 2. Materials and Methods

This study was conducted using a Varian ProBeam proton system. An in-house 3D PBS dose rate calculation tool using pencil beam convolution superposition (PCS) algorithm [30] was developed to calculate the dose rate. The PBS spot delivery time and scanning time between spots were modeled. Similar to the dose volume histogram (DVH) representation of a 3D dose distribution, the 3D dose rate distribution is concentrated using a single dose rate volume histogram (DRVH) curve to represent the voxel-based dose rate distribution. The DRVHs for both OARs and targets were calculated. 

In a Varian ProBeam system, the cyclotron beam current is variable for different energy layers and automatically determined by the minimum monitor unit (MU) of a spot in the energy layer [20]. Thus, the minimum MU/spot of an energy layer determines the deliverable dose rate of the transmission plan. The Varian research group assumed a 2 ms spot delivery time of the minimum MU and 10 mm/ms scanning speed for the ADR dose rate calculation in the ProBeam FLASH mode [20]. The transmission FLASH-RT plans were generated based on the above hypotheses for the ProBeam system. This study was conducted under institutional review board (IRB) approval.

### 2.1. FLASH-RT Treatment Planning

Based on the open-source matRad TPS [31], a planning platform developed in house was used to optimize all transmission IMPT plans using 240 MeV proton beams. The 240 MeV is the highest energy calibrated in TPS; assuming that the 240 MeV beam has a similar spot profile and stopping power in the plateau region compared to 250 MeV beam but having a lower transmission efficiency. Thus, the theoretical transmission of the 250-MeV beam was used for the dose rate calculation. All three methods, DADR, ADR, and DTDR, were applied to derive the DRVHs for all the FLASH-RT plans to assess the dose rate to both tumor and OARs.

**Figure 1:**
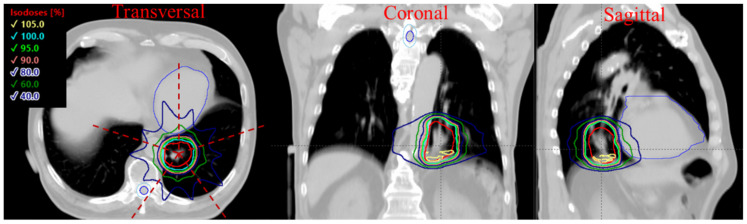
The 3D dose distribution of transmission plan using a 5-field arrangement. The dashed lines indicate the beam direction, and the red contour shows the iCTV.

A cohort of 9 consecutive lung cancer patients previously treated by proton SBRT was replanned using transmission FLASH beams for the study. The transmission plans were developed with two different standard-of-care prescriptions: 45 Gy in 3 fraction and 34 Gy in 1 fraction for all patients [32,33]. Since the short beam-on time of each treatment field (<1 s), the dose interplay effect [34] may not be a concern for each treatment field, while in between fields and fractions, the target motion still needs to be considered. Thus, 4 DCT and the internal target method were used to compensate for the inter-fractional and intra-fractional tumor motion. The internal clinical target volume (iCTV) was generated on an averaged-CT by the union of CTVs on the corresponding 10-phase images of a 4 DCT. The iCTV volume varied from 24 to 226 cm^3^ with a median value of 61 cm^3^. All plans were generated using a 5-beam arrangement with 72 degrees equal angle intervals to give uniform dose distributions to the target. Figure 1 shows the dose distribution and beam arrangement for a typical case, with target coverage for all cases normalized to 100% iCTV receiving 95% of the prescription dose for comparison purposes. The higher the minimum MU/spot in an energy layer, the higher the SPDR can be achieved. The minimum 100 and 400 MU/spot, representing medium and high dose rates, were chosen as the threshold for treatment planning. In total, 36 transmission FLASH-RT plans were optimized for this study.

### 2.2. PBS Dose Rate Calculation Methods

#### 2.2.1. Dose-Averaged Dose Rate (DADR) 

Recently, van de Water et al. [18] proposed a dose-averaged dose rate (DADR) method to evaluate the dose rate on proton PBS planning and treatment applications for head and neck patients. Based on the van de Water et al. method, here we calculate the DADR dose rate in Equation (1).

(1) $${\dot{D}}_{j}^{DADR}=\overset{N}{\underset{i=1}{\sum }}\frac{D_{j,i}}{\sum_{i=1}^{N}D_{j.i}}{\dot{D}}_{j.i}$$

Here, *i* denotes a spot, *j* represents a voxelized region in the target, and $D_{j,i}$ is the dose deposited by the *i*-th spot to the *j*-th voxel. ${\dot{D}}_{j.i}$ is the *i*-th spot dose rate in the *j*-th voxel, which is equivalent to the combination of the proton flux rate and proton dose contribution to the *j*-th voxel, as reported in [18], and geometrically, it is determined by a Gaussian distribution

(2) $${\dot{D}}_{j.i}={\dot{D}}_{max}e^{-\frac{{\left(r_{j}-r_{i}^{c}\right)}^{2}}{\sigma^{2}}}$$

${\dot{D}}_{max}$ is the max dose rate at the spot center that determines the ${\dot{D}}_{j,i}$ following the Gaussian falloff in the spot lateral direction. Here, $r_{j}$ denotes the position of *j*-th voxel, $r_{i}^{c}$ denotes the position of the *i*-th spot center, and 𝜎 is the spot sigma. In this case, to calculate the overall dose rate in a particular voxel j, dose rates contributed from multiple spots are considered during the beam delivery. This method does not account for the temporal separation between spots. Therefore, it will provide the same dose rate estimate from an array of spots, regardless of the duration required to accumulate the dose. 

#### 2.2.2. Averaged Dose Rate (ADR)

Folkerts et al. [20] proposed an averaged dose rate (ADR) method to calculate the PBS dose rate. Both duration of individual spot delivery and scanning from one spot to the next spot are considered for dose rate calculation. The dose rate calculation formula is shown in Equations (3)–(6) for a particular voxel, *j*; ($D_{j}$ − 2d*) is the total dose deposited in voxel *j* during the irradiation $T_{j}$, $d^{*}$ is a preset dose-threshold that determines the irradiation start time $t_{0}$ and the end time $t_{1}$. By applying the dose-threshold, $d^{*}$, the non-significant dose accumulation to voxel j from all the scanning spots is excluded from the dose rate calculation. A dose-threshold of 0.1 Gy was chosen as the cutoff by Folkerts et al. This dose rate value can be calculated for voxels in a region-of-interest, and statistics reported accordingly.

(3) $${\dot{D}}_{j}^{ADR}=\frac{D_{j}-2d^{*}}{T_{j}}$$

where,

(4) $$d_{j}\left(t_{0}\right)=d^{*}$$

(5) $$d_{j}\left(t_{1}\right)=D_{j}-d^{*}$$

(6) $$T_{j}=t_{1}-t_{0}$$

#### 2.2.3. Dose-Threshold Dose Rate (DTDR)

PBS delivers doses via spot-scanning. The dose is at its maximum at the spot center and decreases from the center to the lateral direction. DTDR uses a dose-threshold to exclude the low dose tails of spots from dose rate calculation. As shown in Equation (7), for voxel j, the dose-threshold dose rate (DTDR) is the minimum instantaneous dose rate of all the spots that deposit dose to the voxel above a predefined dose-threshold $d^{*}$, ${\dot{D}}_{j,i}$ is the *i*-th spot dose rate in the *j*-th voxel calculated using Equation (2).

(7) $${\dot{D}}_{j}^{DTDR}=\min\left({\dot{D}}_{j,i}\right),\;\mathrm{if}\;D_{j,i}>d^{*},\;i=1,2\ldots n$$

### 2.3. Pencil Beam Scanning Parameters 

**Figure 2:**
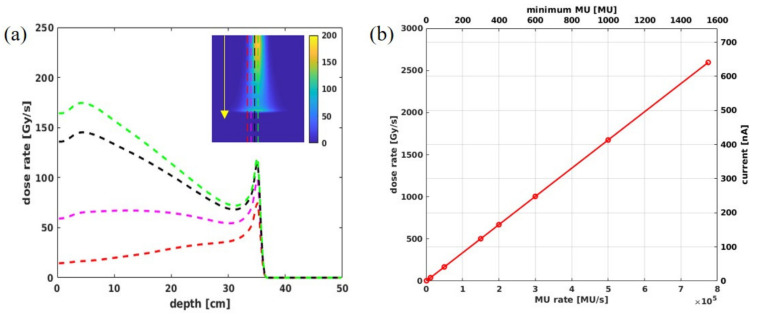
(**a**) The dose rate profile for a 240 MeV single spot with 100 MU at the central plane along depth direction (indicated by the arrow), (**b**) the theoretical calculation for nozzle current, minimum MU/spot, SPDR, and MU for proton beam under FLASH mode.

Monte Carlo beam modeling of the Varian ProBeam system has demonstrated that the definition of 1 MU contains ~5.17 × 10^6^ protons for 240 MeV beams [35]. Figure 2a reflects the dose rate distribution for a single spot with 100 MU in a water phantom. Figure 2b shows the correlations of SPDR, nozzle beam current, and MU/s for the FLASH mode. Theoretically, when the system works at the highest transmission with a nozzle current ~640 nA [36,37,38], it corresponds to an SPDR of 2600 Gy/s. This study used minimums of 100 and 400 MU/spot, corresponding to two different instantaneous dose rates of 167 Gy/s and 670 Gy/s, to assess the hypofractionation lung transmission FLASH plan quality and 3D volume dose rate distribution using these three types of dose rate metrics.

## 3. Results

### 3.1. Phantom Dose Assessment 

**Figure 3:**
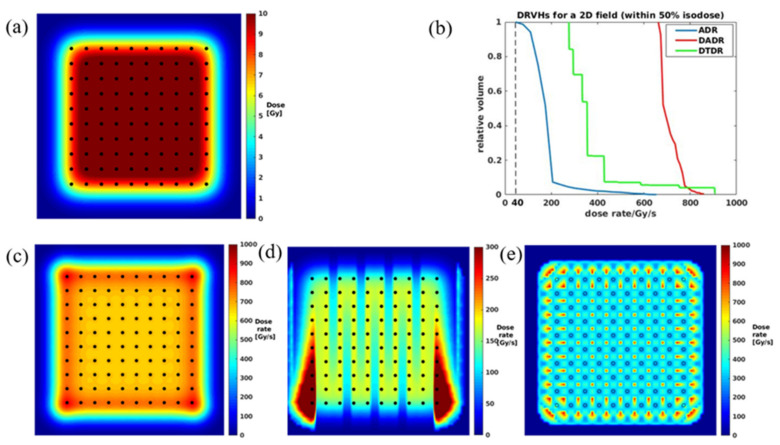
(**a**) A 5 × 5 cm^2^ dose map at water phantom surface with spots marked by dots, (**b**) DRVH comparisons for all three calculation methods, (**c**–**e**) 2D dose rate distributions for DADR, ADR, and DTDR, respectively.

To benchmark our calculation with Folkerts et al. [20], we created the same spot map and set the SPDR to 1300 Gy/s. As shown in Figure 3a, a 5 × 5 cm^2^ field with a spot spacing of 5 mm was generated in a water phantom. All three different dose rate calculations are performed, as shown in Figure 3c–e, and the color wash indicates the magnitude of the dose rate. Figure 3b shows DRVHs for the three methods, demonstrating that the DADR, ADR, and DTDR methods calculated the dose rate in the same phantom, and all three dose rate methods could result in 100% of the volume being covered by at least 40 Gy/s. Because the ADR method considers each spot’s duration and scanning time, it gives the lowest dose rate compared to DADR and DTDR methods. Additionally, we observed from Figure 3d that the ADR is scanning direction-dependent, and there are two dose rate bump strips at the most outside spot lines of the scanning map. The DADR method weighted the dose without considering the spot duration and scanning time; the dose rate was uniform across the scanning map with the highest dose rate in most outside spots. The DTDR method uses a 0.1 Gy dose-threshold to exclude the spot low dose tail from the dose rate calculation, making the dose rate distribution a regular pattern. This dose rate pattern is similar to the spot map, but the highest DTDR occurs between the spots not at the spot center, which is different from the results of the ADR method. This can be explained as the spot dose, and dose rate follows a Gaussian dose rate falloff from the spot center to the surrounding area, the lower dose rate at the spot center originates from the adjacent spots; DTDR of a voxel is the minimum dose rate contributed by the neighboring spot; therefore, the 2D dose rate distribution shows a lower dose rate at the center of a spot. On the other hand, the dose-threshold determines the distance from the region-of-interest to the adjacent spots that can be considered for dose rate calculation. For instance, if a spot center dose is 100%, the dose reduces to 1% at ~3 × sigma from the spot center which corresponds to a dose rate of 1% of the spot center max dose rate at a distance of ~3 × sigma. Thus, a large dose-threshold will generate a higher DTDR distribution and vice versa.

### 3.2. Plan Quality Assessment 

**Figure 4:**
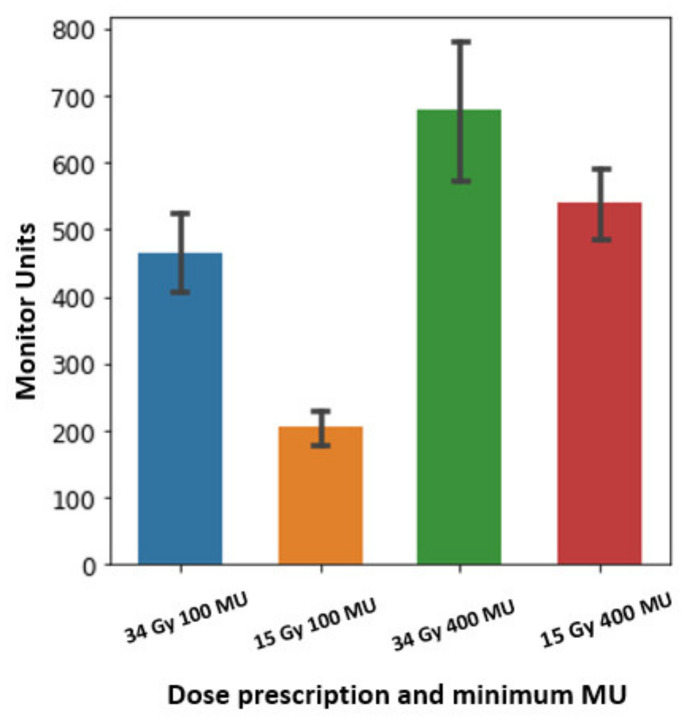
The MU statistics of spots for one patient using two different prescriptions (15 Gy × 3 fractions and 34 Gy × 1 fraction) and two minimum MU/spot settings: the bar height represents the mean MUs of one plan. The error bar represents the MU standard deviation from the mean values.

Figure 4 shows the MU distributions of all spots for one patient. The mean MU/spot is ~200 for the 15 Gy-100 MU plan, while the mean MU/spot is 455 MU for the 34 Gy-100 MU plan. For the 15 Gy-400 MU plan, the mean MU/spot is 530 MU, while for the 34 Gy-400 MU plan, the mean MU/spot is ~660 MU. 

**Figure 5:**
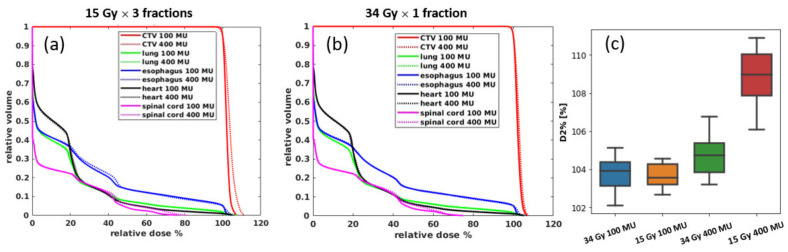
The average DVHs for all 9 patients for (**a**) 15 Gy × 3 plans and (**b**) 34 Gy × 1 plans; (**c**) is the target uniformity comparison using D_2_% as the representative for the hot dose; the ends of the box denote the interquartile (25–75th percentile), a horizontal line inside the box marks the median, and the whiskers are the two lines outside that represent the highest and lowest observation.

All transmission plans could achieve a reasonably good uniformity (hotspot < 110% of prescription dose). Figure 5a,b shows the averaged DVHs of the target volumes and OARs for all nine patients. The 100 MU plan had a better uniformity and lower OAR doses for both of the different prescriptions. As shown in Figure 5c, the D_2_%, representing the hot spots dose volume, can be reduced by ~5% in the 15 Gy plans using 100 MU/spot instead of 400 MU/spot, whereas, for the 34 Gy plan, the hot dose difference is minimal between two different MU/spot settings. This indicates that the plan with a higher fraction dose (34 Gy/fraction) could have better uniformity by using both low and high (100 and 400) MU/spot, whereas the lower fraction dose (15 Gy/fraction) plan needs a smaller minimum MU/spot to achieve a more uniform dose distribution.

Table 1 presents the OAR dose metrics of the four scenarios for the spinal cord, functional lung, heart, and esophagus, and all these mean values and their standard deviations were derived based on the average DVHs for all nine patients. As no constraints are available for FLASH-RT yet, constraint parameters from RTOG0915 for 34 Gy in one fraction SBRT are used [32,33]. Both 100 and 400 MU/spot plans are compared; in general, lower MU plans can achieve lower OAR dose, especially for 15 Gy in three-fraction plans, while for 34 Gy in one-fraction plans, the dosimetry differences are small between 100 and 400 MU/spot settings.

### 3.3. 3D Dose Rate

**Figure 6:**
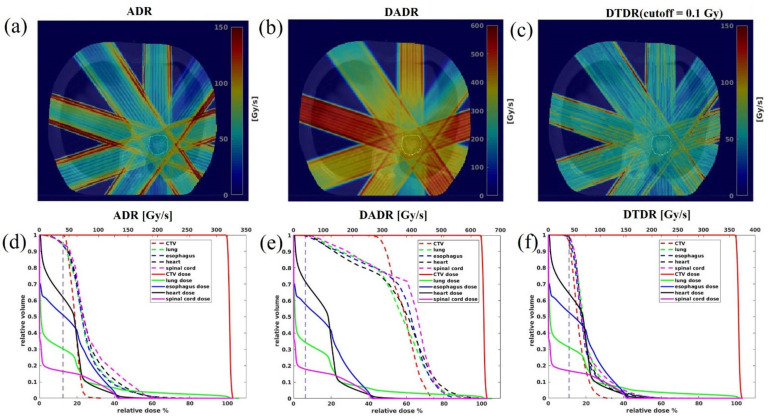
The dose rate comparison among all three methods for a typical patient using a minimum 400 MU/spot and 34 Gy × 1 fraction. (**a**–**c**) Show the dose rate distributions from each different field over patient anatomy, (**d**–**f**) are the DVHs and DRVHs, the solid lines are DVHs and the dashed lines are the DRVHs, and the 40 Gy/s dose rate is indicated by the vertical dashed lines.

The dose rate distribution was calculated for each field then overlaid on the CT images. Therefore, the voxels having non-zero doses for each field were included for DRVH calculation, and the DRVH of a plan was sampled from all five fields. Figure 6a–c shows the 3D dose rate distributions over the patient anatomy for each beam angle, calculated using ADR, DADR, and DTDR methods. As seen in Figure 6a–c, the high dose rate regions often occurred at the field edge for ADR and DTDR, while DADR resulted in a better dose rate uniformity over the whole field. All three dose rates attenuated along the beam direction due to the protons being scattered gradually when passing through tissue. Figure 6d–f shows the DVHs and DRVHs for the target and OARs; as shown in Figure 6d, a notable portion of OARs received irradiation at dose rates below 40 Gy/s dose rate threshold when using the ADR method, whereas the majority of the volume of each OAR received irradiation with a dose rate > 40 Gy/s when using the DADR and DTDR methods.

**Figure 7:**
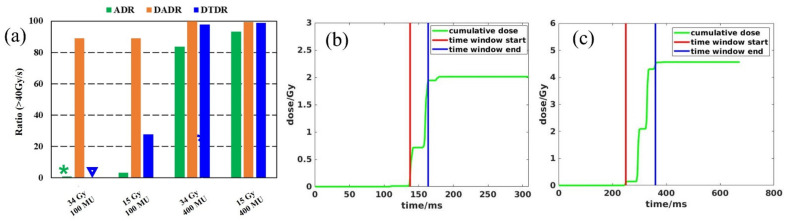
(**a**) is the averaged V_40Gy/s_ for all the OARs under each of the three dose rate methods; the ADR and DTDR are 1.0% and 0.3% are represented by a star and triangle for better illustration. (**b**,**c**) Time structures determined by dose-threshold 0.1 Gy for ADR calculation under 15 Gy/fraction and 34 Gy/fraction, respectively.

To quantify the target dose rate and OARs to determine if the FLASH dose rate was reachable, a dose rate coverage index V_40Gy/s_ was defined, representing the percentage of the volume receiving a dose rate ≥ 40 Gy/s. Table 2 presents the dose rate statistics among the three different calculation methods for all nine patients. Not surprisingly, ADR considering the spot dwelling and scanning time gave the lowest dose rate, DTDR using instantaneous dose rate not considering any other time effect but a dose-threshold of 0.1 Gy gave an intermediate dose rate, and DADR gave the highest dose rate without considering any time or dose thresholds. The SPDR for 100 MU/spot was ~168 Gy/s, the ADR V_40Gy/s_ of iCTV for 34 and 15 Gy/fraction were only 0.04% and 0.3%, and when using 400 MU/spot with SPDR of ~670 Gy/s, the ratios increased to 81.4% and 99.3%. DADR could always maintain a desired V_40Gy/s_ of iCTV (~100%) using either a minimum 100 or 400 MU/spot for 15 or 34 Gy/fraction plans. When the dose rate was calculated using DTDR, V_40Gy/s_ of iCTV was much higher for 400 MU/spot plans than the 100 MU/spot plans. In addition, the V_40Gy/s_ for 15 Gy/fraction was higher than the 34 Gy/fraction plans for both 100 and 400 MU/spot settings. Figure 7a summarizes the averaged dose rate for all the OARs under each of the planning scenarios, and similar trends of V_40Gy/s_ for iCTV can be found for the OARs. The ADR could not give sufficient OAR V_40Gy/s_ coverage using 100 MU/spot for 34 or 15 Gy/fraction plans; however, the V_40Gy/s_ could be increased to 83.6% (34 Gy/fraction) and 93.4% (15 Gy/fraction) when a 400 MU/spot was used. Similar to the target dose rate, DADR always calculated a high OARs V_40Gy/s_ (>88.9%). In the DTDR method, the V_40Gy/s_ of OARs was much higher under a 400 MU/spot than under a 100 MU/spot. Meanwhile, the V_40Gy/s_ for 15 Gy/fraction plans was higher than the 34 Gy/fraction plans. Compared to a 15 Gy/fraction plan, the spots at larger distances for the 34 Gy/fraction plan were also considered to contribute to the local instantaneous dose rate when applying an absolute dose threshold in DTDR due to its higher mean spot dose. Each spot had the same machine-defined SPDR and a dose rate falloff following a Gaussian distribution. The inclusion of distant spots guaranteed a lower local instantaneous dose rate. 

## 4. Discussion

Thoracic malignancies are particularly challenging tumors to treat and are associated with, perhaps, the highest rates of radiation-induced high-grade toxicities. Definitive radiation therapy for lung and thoracic cancers can result in potentially fatal radiation pneumonitis, quality-of-life-limiting pulmonary fibrosis, and esophagitis that can lead to hospitalizations and failure to thrive [39]. Additionally, there is increasing recognition that radiotherapy to the chest can lead to a variety of cardiac toxicities and major cardiac events [40]. As such, ways to reduce both acute and late toxicities associated with thoracic radiotherapy are critically needed. The potential normal tissue sparing effect of FLASH might be a particularly attractive clinical option. This study investigated three types of 3D dose rate calculation methods and quantified their difference by assessing the dose rates for nine lung cases under two prescribed hypofractionation treatment scenarios. The received dose rate for OARs is one of the major considerations in the FLASH sparing effect; however, the entire OAR volume in the beam path was not always found to reach the FLASH dose rate (>40 Gy/s). The differences in dose rate metrics have brought a large variation in DRVH for OARs and targets. The realistic parameters of minimum MU/spot and fractionation dose need to be optimized to achieve sufficient plan quality and a high ratio of coverage for OARs with FLASH dose rate.

The MU definition varies between different vendors, and the spot delivery mechanism can also be different. ProBeam working under a layer-wise delivery manner means that the spot dose rate of each layer is determined by the minimum MU/spot. Awareness of the delivery mechanism difference between different types of machines will be important to model the dose rate correctly. This study was based on a Varian ProBeam system, and the other types of spot delivery mechanisms were not included. 

The clinical cyclotron systems, except for one compact synchrocyclotron system using range shifter plates in treatment nozzle to pull back proton ranges [41], all use energy degrader and energy selection system to generate lower energy proton beams to treat tumors at variable depths. The energy selection and beam trimming by apertures cause a large number of protons to be lost in the proton transportation. The low transmission efficiency for lower energy beams prevents it from achieving a higher beam current in the treatment room. Additionally, the energy switch time (~200 ms) is relatively long compared to the total field delivery time of <1 s. Therefore, the transmission plans with high-energy beams are more suitable for FLASH-RT applications. However, the transmission plans do not use any Bragg peak for dose delivery, which results in exposure to normal tissues distal to the target volume and unnecessary irradiation exposure not seen with clinical proton therapy [42]. 

The first proton PBS human trial is using a forward treatment planning method [43], and currently, the accessibility of commercial TPSs for FLASH inverse treatment planning is limited. The TPS capability to optimize the spot weight may vary between different TPSs. Different optimizers and dose calculation engines (analytical versus Monte Carlo algorithm) can result in various plan quality and dose rate distributions. The planning parameters, including the minimum MU/spot, fraction dose, selection of beam angles, prioritization for OARs sparing, and target coverage, all impact the plan quality and final dose rate distribution. Therefore, the treatment planning strategies, the DVH, and DRVH need to be extensively studied once these commercial TPSs are available for clinical application.

The duration of each spot is at an order of magnitude ~10^−3^ s, the radiation-induced events like the DNA damage begin over a time scale of 10^−12^–10^−7^ s [44,45], the rapid consumption of local oxygen occurs on the scale of 10^−3^ s [46], and oxygen diffusion in a time-scale of ~10^−2^ s [47]. Different researchers have proposed that either the mean dose rate over the entire field delivery time [12,22,24] or the instantaneous dose rate of the pulse [21] is more relevant to the FLASH sparing effect based on their understanding of the currently available experimental data. DADR and DTDR, using the instantaneous dose rate, ignore the dwelling time of spots and scanning time between spots which may overestimate the dose–rate effect. The dose–threshold of DTDR could be tissue-specific, and the meaning of the values is not clear yet. Adrian et al. [48] conducted the in vitro FLASH-RT versus conventional-RT experiments to characterize the correlation between cell survival fraction and the delivered dose, dose rate, and oxygen concentration. This in vitro analysis showed that for a particular hypoxic condition (1.6% oxygen concentration), the FLASH-sparing effect starts at 5–10 Gy, is apparent at ≥15 Gy, and significant at 18 Gy. Earlier studies by Wilson et al. [49] have shown that for oxygen concentrations of 0.4%, a dose of 5–10 Gy is sufficient to deplete cellular oxygen at the FLASH dose rate. Adrian et al. [48] studies show no survival fraction difference for dose < 5 Gy between FLASH-RT and conventional-RT. Different values of the dose-threshold will produce a wide deviation of the DTDR distribution. Alternatively, ADR averages dose rates over the entire delivery of one field, giving lower dose rate estimation. Similarly, the dose-threshold of 0.1 Gy used in the ADR method is debatable. A clinically relevant dose-threshold needs to be determined by the biological study. In addition to minimum MU/spot and dose-threshold, the fraction dose also plays an important role in determining the dose rate distribution. Figure 7a shows that the 34 Gy/fraction plans have a lower ADR than the plans with lower fractional dose of 15 Gy. This can be explained by applying the preset dose–threshold 0.1 Gy in the ADR calculation. As indicated in Figure 7b,c, for an arbitrary voxel, the 0.1 Gy threshold determines the start and end time window for the ADR calculation. For a given dose rate (MU/s), the 15 Gy/fraction plan has a much narrow time span than the 34 Gy/fraction plans since the mean MU/spot of 34 Gy/fraction is larger than the 15 Gy/fraction plans. The ADR takes both the spot and the scanning time into consideration, and for a given cyclotron beam current or minimum MU/spot, a larger field size requires a longer beam time; therefore, the dose rate and the V_40Gy/s_ coverage will decrease with the increase of field size. 

If it is possible, it will be important to quantify proton PBS dose rate using these 3-metric during pre-clinical study for better understanding the roles of the dose rate in FLASH sparing effect. Recent research [50] using both continuous (40 Gy/s) and pulsed beams (40 Gy/s with 10% duty cycle) studied the FLASH efficacy for the treatment of non-small cell lung cancer in mice. That study found that both FLASH and Pulsed-FLASH dose-rate modes had significantly smaller lung tumors than mice treated with proton radiation delivered at conventional dose-rate. It will be of great interest to understand the roles of the instantaneous versus the time-averaged dose rate when more data are available to the FLASH-RT community. The limitation has been pointed out by other researchers [18], namely that “all the pre-clinical FLASH experiments only using single beams and with a validated radiobiological explanation for the FLASH effect still lacking, the impact of dead times between spots on the effective dose rate remains unclear.” Additional in vitro and in vivo work is needed to determine if the FLASH effect is still achieved as fractionation increases and the number of fields increases.

## 5. Conclusions

The minimum MU/spot settings are critical to maintain an acceptable plan quality while at the same time reaching a FLASH dose rate. The higher MU/spot corresponds to the higher dose rate, while the target uniformity becomes worse. The plans with higher fraction doses have better target uniformity than the ones with lower fraction doses when the minimum MU/spot is higher (400 MU/spot). The ADR and DTDR methods show that part of OARs could not reach the FLASH dose rate, whereas the DADR method gives a much higher dose rate than the ADR and DTDR methods. The different PBS dose rate calculation methods may result in a different correlation expectation between dose rate metrics and biological effects for pre-clinical experiments. An awareness of the differences in proton PBS dose rate calculation is important to design experiments and clinical trials to uncover FLASH-RT’s biological and physiological mechanisms.

## Figures and Tables

**Table 1:** OAR dose metrics for all transmission plans (RTOG0915 metrics are applied).

OAR	RTOG0915 Constraints	34 Gy × 1 (100 MU)	34 Gy × 1 (400 MU)	15 Gy × 3 (100 MU)	15 Gy × 3 (400 MU)
Esophagus	D 5 cc (Gy)	19.1 ± 9.8	19.5 ± 9.8	24.2 ± 14.2	25.9 ± 12.1
	D max (Gy)	25.2 ± 9.5	25.5 ± 9.3	33.3 ± 12.1	34.8 ± 11.6
Heart	D 15 cc (Gy)	13.8 ± 14.1	13.6 ± 13.8	17.5 ± 18.4	18.2 ± 18.6
	D max (Gy)	20.9 ± 14.6	21.3 ± 14.2	26.6 ± 18.6	28.6 ± 19.2
Lung-GTV	V 7 Gy (cc)	909.2 ± 382.2	924.3 ± 399.2	1111.1 ± 495.4	1178.8 ± 495.0
	V 7.4 Gy (cc)	825.0 ± 348.0	833.8 ± 370.4	1081.6 ± 495.9	1162.8 ± 488.9
Spinal cord	D 0.35 cc (Gy)	17.4 ± 5.1	17.9 ± 5.4	22.7 ± 7.5	25.4 ± 8.0
	D 1.2 cc (Gy)	16.3 ± 4.6	16.9 ± 5.0	21.4 ± 6.9	24.0 ± 7.6
	D max (Gy)	18.7 ± 5.7	19.1 ± 5.8	24.3 ± 8.3	27.6 ± 8.8

**Table 2:** V_40Gy/s_ statistics for iCTV and OARs among ADR, DADR, and DTDR methods. The ratios are calculated by averaging the plans for all nine patients under each of the three dose rate methods, and the standard deviation is expressed in the parentheses. The last row of the table shows the averaged dose rate for the five OARs under each of the three dose rate methods for illustration purposes.

	34 Gy × 1, Min MU: 100MU			15 Gy × 3, Min MU: 100MU			34 Gy × 1, Min MU: 400MU			15 Gy × 3, Min MU: 400MU		
	ADR	DADR	DTDR	ADR	DADR	DTDR	ADR	DADR	DTDR	ADR	DADR	DTDR
					(%)							
iCTV	0.04 (0.1)	98.8 (2.5)	0.0 (0)	0.3 (0.8)	98.8 (2.5)	25.7 (12.3)	81.4 (23.1)	100 (0.03)	97.2 (2.4)	93.3 (7.4)	100.0 (0.13)	99.9 (0.2)
esophagus	0.8 (1.6)	91.4 (5.7)	0.3 (0.7)	3.1 (1.5)	91.0 (5.5)	26.7 (15.1)	87.4 (10.4)	99.8 (0.2)	97.6 (3.4)	94.2 (2.5)	99.7 (0.21)	99.1 (0.61)
heart	1.3 (2.7)	82.9 (11.9)	0.4 (0.5)	3.1 (1.7)	83.5 (11.9)	28.2 (15.4)	79.1 (13.5)	99.9 (0.18)	97.1 (3.0)	91.6 (5.0)	98.8 (0.84)	98.4 (1.4)
lung	1.2 (1.84)	92.1 (3.8)	0.2 (0.2)	3.6 (1.8)	91.5 (3.8)	31.1 (13.0)	87.4 (10.6)	100 (0.07)	98.2 (1.6)	94.2 (2.9)	99.5 (0.27)	99.4 (0.22)
spinal cord	0.7 (1.1)	89.3 (3.4)	0.3 (0.8)	3.1 (2.3)	90.1 (3.6)	24.7 (14.8)	80.6 (16.4)	99.9 (0.14)	98.5 (1.8)	93.5 (3.0)	99.4 (0.46)	98.8 (0.96)
AVG_ OARs	1.0	88.9	0.3	3.2	89.0	27.7	83.6	99.9	97.9	93.4	99.4	98.9

## Data Availability

The data presented in this study are available on request from the corresponding author.
